# Lung function and quality of life one year after severe COVID-19 in Brazil

**DOI:** 10.36416/1806-3756/e20230261

**Published:** 2024-05-08

**Authors:** Tarciane Aline Prata, Arnaldo Santos Leite, Valéria Maria Augusto, Daniel Cruz Bretas, Bruno Horta Andrade, Jaqueline das Graças Ferreira Oliveira, Aline Priscila Batista, George Luiz Lins Machado-Coelho, Eliane Mancuzo, Carolina Coimbra Marinho

**Affiliations:** 1. Universidade Federal de Minas Gerais - UFMG - Belo Horizonte (MG) Brasil.; 2. Hospital Eduardo de Menezes - Fundação Hospitalar de Minas Gerais - FHEMIG - Belo Horizonte (MG) Brasil.; 3. Universidade Federal de Ouro Preto - UFOP - Ouro Preto (MG) Brasil.

**Keywords:** COVID-19, Respiratory function tests, Spirometry, Dyspnea, Quality of life

## Abstract

**Objective:**

To evaluate symptoms, lung function, and quality of life of a cohort of patients hospitalized for severe COVID-19 12 months after hospital admission.

**Methods:**

This was a cross-sectional study. We included severe COVID-19 survivors hospitalized in one of three tertiary referral hospitals for COVID-19 in the city of Belo Horizonte, Brazil. Participants were submitted to lung function and six-minute walk tests and completed the EQ-5D-3L questionnaire.

**Results:**

The whole sample comprised 189 COVID-19 survivors (mean age = 59.6 ± 13.4 years) who had been admitted to a ward only (n = 96; 50.8%) or to an ICU (n = 93; 49.2%). At 12 months of follow-up, 43% of patients presented with dyspnea, 27% of whom had a restrictive ventilatory disorder and 18% of whom presented with impaired DL_CO_. There were no significant differences in FVC, FEV_1_, and TLC between the survivors with or without dyspnea. However, those who still had dyspnea had significantly more impaired DL_CO_ (14.9% vs. 22.4%; p < 0.020) and poorer quality of life.

**Conclusions:**

After one year, survivors of severe COVID-19 in a middle-income country still present with high symptom burden, restrictive ventilatory changes, and loss of quality of life. Ongoing follow-up is needed to characterize long COVID-19 and identify strategies to mitigate its consequences.

## INTRODUCTION

COVID-19, caused by SARS-CoV-2, has been acknowledged to be responsible for a multisystemic disorder.^1^ Similarly to other coronaviruses, there are also reports of prolonged symptoms after COVID-19.^2^

There are various mechanisms that may be involved in symptom persistence.^1^ ^,^ ^3^ In a prospective cohort from Wuhan, China, dyspnea was reported in 26% of patients after 6 months.^4^ Interstitial abnormalities were observed in 55.7% of patients after a mean of 90 days from hospital discharge.^5^ A reduced DL_CO_ has been the most frequently detected alteration in the long term.^6^ Scientific studies comparing clinical data and pulmonary function after 45 days or 3 months and 6 months of hospitalization for severe COVID-19 showed that there was improvement in pulmonary function at 6 months.^4^ ^,^ ^7^ ^-^ ^9^

Physical fitness deficit was associated with dyspnea and fatigue in studies of persistent symptoms after COVID-19.^10^ ^,^ ^11^ However, in a study that evaluated six-minute walk test (6MWT) results after hospital discharge and then again after 3 months, no differences were found in demographic, anthropometric, physiological, and clinical characteristics or in the perception of health status between patients with and without exercise limitation.^12^

Poor quality of life (QoL) has been detected in 59% of 1,108 participants pooled in a systematic review and meta-analysis with survivors of COVID-19.^13^ Another review, including only studies involving hospitalized patients, identified that COVID-19 patients had worse health-related QoL (HRQoL) when compared with hospitalized patients without COVID-19.^14^

The aim of this study was to describe alterations in lung function and perceived HRQoL in a cohort of patients 1 year after hospital admission for severe COVID-19 in Brazil and to compare COVID-19 patients who were admitted to a ward only and those admitted to an ICU.

## METHODS

This is a nested cross-sectional study in a multicenter cohort of COVID-19 survivors evaluating patients 12 months after admission to one of three public referral hospitals for COVID-19 in the city of Belo Horizonte, Minas Gerais, Brazil, namely, *Hospital das Clínicas da Universidade Federal de Minas Gerais*, *Hospital Júlia Kubitschek*, and *Hospital Eduardo de Menezes*, between May 25, 2020 and December 28, 2020, during the first wave of COVID-19. During that period, vaccination was unavailable in the country. Patients were stratified into two groups: patients admitted only to a ward, that is, who never required admission to an ICU; and patients admitted to an ICU, who required high-flow oxygen therapy, mechanical ventilation, or use of vasopressors during ICU stay.

Patients ≥ 18 years of age who had ARDS upon hospital admission were included. COVID-19 was confirmed by a positive RT-PCR result of a nasal swab sample. A case of ARDS was defined as an individual with fever and cough or sore throat, associated with dyspnea, chest tightness, or Spo_2_ < 95%.^15^ Eligible patients at hospital admission were invited to participate in outpatient follow-up and were included in the study when they attended the outpatient clinic 360 days after admission and completed the study protocol. Patients who withdrew consent were excluded from the analysis.

The study was approved by the Brazilian National Research Ethics Committee under protocol number 5.416.966. All participants were invited to participate in the study and were included after the participant signed an informed consent form.

Demographic data, clinical manifestations, comorbidities, continuous medication, smoking, date of respiratory symptom onset, date of hospital admission, length of hospital stay, length of ICU stay, duration of mechanical ventilation, and complications during hospitalization were recorded.

During consultations at the outpatient clinics, information on the participants’ QoL was collected using the EQ-5D-3L questionnaire (EuroQoL Research Foundation).^16^ The instrument consists of a descriptive form, comprising five dimensions: mobility, self-care, usual activities, pain/discomfort, and anxiety/depression. For each dimension there are three levels: no problems, moderate problems, and extreme problems. Patients were asked to indicate their health status by checking the box next to the most appropriate statement in each of the five dimensions. Finally, the patient assigned a value to their QoL using a visual analog scale from 0 (worst health) to 100 (best health).

The main outcomes studied were lung function (spirometry, lung volumes, and DL_CO_), physical exercise capacity measured by the distance covered in six minutes (6MWD), respiratory muscle strength (MIP and MEP), and perceived QoL at 12 months after hospital admission.

According to the WHO, the post-COVID-19 condition occurs in individuals with a history of probable or confirmed SARS-CoV-2 infection, generally 3 months after the onset of COVID-19, with symptoms lasting at least 2 months that cannot be explained by alternative diagnoses.^17^

At follow-up, the persistence of cough and dyspnea (according to the modified Medical Research Council scale),^18^ vital data, weight, and height were recorded. Lung function tests were performed in the Pulmonary Function Laboratory of the University Hospital of the Federal University of Minas Gerais. Lung volumes were measured using a Vyntus^TM^ body plethysmograph (Vyaire Medical Inc., Höchberg, Germany) of variable pressure equipped with a pneumotachograph in accordance with the standards proposed by the American Thoracic Society and the European Respiratory Society.^19^ ^,^ ^20^ The following variables were studied: TLC, slow vital capacity, FVC, FEV_1_, and the FEV_1_/FVC ratio. Measurements were expressed in absolute values and in percentage of predicted (%pred) values for the Brazilian population.^21^ ^,^ ^22^ The single breath method was used to determine DL_CO_, considering the values suggested by Guimarães et al.^23^

The 6MWT was performed in a 30-m corridor using a portable oximeter (Nonin Medical Inc, Plymouth, MN, USA) according to international recommendations.^24^ The following variables were recorded: oxygen saturation (Spo_2_), heart rate (HR), respiratory rate (RR), dyspnea score on the Borg scale at the beginning and end of the 6MWT, HR in %pred relative to the maximum HR in %pred for adults, HR at the end of the 6MWT, HR after 1 min of recovery from the 6MWT (HRR_1_), and 6MWD. A fall in oxygen saturation ≥ 4% or a reduction in HR after 1 min of 6MWT recovery < 12 bpm were considered altered results.^24^ The 6MWD was expressed in absolute values and in %pred for the Brazilian population.^25^

MIP and MEP were measured with an analog manometer (Makil, Londrina, Brazil) as described by Laveneziana et al.^26^ The maneuver was repeated five to eight times, respecting a reproducibility of 10%. The highest value obtained was recorded. The predicted values were calculated according to Neder et al.^27^ The lower limit of normality (LLN) for each variable was calculated from predictive equations.^20^

Possible sources of bias were the diagnosis of COVID-19, lung function measurements, and selection bias. Diagnosis was defined by RT-PCR; the equipment was calibrated according to recommendations of the manufacturers, and clinical evaluation was based on standardized questionnaires. Selection bias was minimized by the multicenter design.

Data were collected using the REDCap platform (Vanderbilt University, Nashville, TN, USA)^28^ and analyzed with the IBM SPSS Statistics software package, version 28.0 (IBM Corporation, Armonk, NY, USA). Categorical variables were described as frequencies and ratios. Continuous variables with normal distribution were described as means and standard deviations, while those with non-normal distribution were described as medians and interquartile ranges. The predicted values and the LLN were used as risk to categorize continuous variables. The parametric Student’s t-test or the nonparametric Mann-Whitney U test were used to check the differences in means and medians, respectively, between groups, and the Pearson’s chi-square test for ratios. Binary logistic regression analysis was used to adjust associations by BMI, use of mechanical ventilation, acute kidney injury, and length of hospital stay. Hypothesis testing was two-sided, and the significance level was set at p < 0.05.

## RESULTS

**Figure 1:**
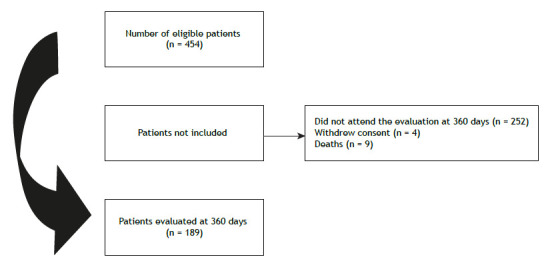
Flow chart of the participant selection process.

At hospital admission during the study period, 454 patients were considered eligible, but 252 did not attend the evaluation at 360 days, 4 withdrew consent, and 9 died. The final sample consisted of 189 patients evaluated 360 days after hospitalization for severe COVID-19 (Figure 1). Among those lost to follow-up, a greater proportion corresponded to patients who had been admitted to the ICU (p = 0,032; Supplementary Table 1)

The Ward and ICU groups were composed of 96 (50.79%) and 93 (49.20%) of participants, respectively. The groups were homogeneous regarding demographic variables: age (59.6 ± 13.4 years), gender (49.2% were male), presence of comorbidities (88.8%), schooling, family income, and self-declared skin color. Among the pre-existing conditions, there was a difference between the groups only regarding the presence of obesity, which was more frequent in the ICU group (p = 0.018). In the sample as a whole, asthma and COPD were reported in 11.1% and 6.7%, respectively, and 26.6% of the patients were smokers (Table 1).

After 12 months, we found persistence of cough and dyspnea in 19% and 43% of patients in the Ward and ICU groups, respectively, but with no statistical difference. In the logistic regression analysis, no significant difference was observed between the groups regarding spirometry variables, lung volumes, DL_CO_, 6MWT, and muscle strength (p > 0.05; Table 2).

After stratifying the patients into two groups, with and without dyspnea, we observed that cough was more common in the first group, as well as higher BMI values. Lung function variables (VC, FVC, FEV_1_, FEV_1_/FVC ratio, TLC, DL_CO_, MIP, and MEP) obtained in the group with dyspnea were significantly lower. However, the frequency of altered variables did not differ significantly between the groups, except for DL_CO_ [(dyspnea: 15 (14.9%) vs. no dyspnea: 17 (22.4%); p = 0.011]. The dyspnea group had shorter 6MWD, with a higher percentage of patients with a final Borg scale score ≥ 4, (44.4% vs. 8.9%; p = 0.001; Table 3).

**Table 1:** Sociodemographic and clinical characteristics, as well as pre-existing conditions, at baseline in the sample as a whole and by group (Ward vs. ICU).^a^

Variable		Total sample	Group		p
			Ward	ICU	
		N = 189	n = 96	n = 93	
Age, years		59.6 ± 13.4	60.9 ± 14.3	58.4 ± 12.4	0.197
Men, n (%)		93 (49.2)	49 (51.0)	44 (47.3)	0.608
Comorbidities^b^		167 (88.8)	85 (89.5)	82 (88.2)	0.777
Variable	Category	n (%)			
Schooling^b^	Undergraduate/graduate education	17 (9.7)	7 (7.8)	10 (11.6)	0.165
	Middle to high school	76 (43.2)	45 (50.0)	31 (36.0)	
	No education or incomplete elementary school (< 8 years)	83 (47.2)	38 (42.2)	45 (52.3)	
Income^b^	> 3 MW	33 (19.4)	18 (20.7)	15 (18.1)	0.633
	< 3 MW	131 (77.1)	67 (77.0)	64 (77.1)	
	No income	6 (3.5)	2 (2.3)	4 (4.8)	
Self-reported skin color^b^	White	56 (29.8)	30 (31.3)	26 (28.3)	0.654
	Non-White^d^	132 (70.2)	66 (68.8)	66 (71.7)	
Prevailing circumstances					
Hypertension^c^		118 (71.1)	56 (66.7)	62 (75.6)	0.204
Obesity^c^		66 (42.0)	28 (33.3)	38 (52.1)	0.018
Diabetes mellitus^c^		53 (31.9)	22 (25.9)	31 (38.3)	0.087
Other cardiovascular diseases^c^		20 (12.4)	11 (13.1)	9 (11.7)	0.787
Asthma^c^		18 (11.1)	10 (11.9)	8 (10.3)	0.739
COPD^c^		11 (6.7)	4 (4.8)	7 (8.9)	0.297
Chronic kidney disease^c^		8 (5.0)	3 (3.6)	5 (6.5)	0.394
Other comorbid disorders^c^		76 (46.3)	43 (50.6)	33 (41.8)	0.258
Smoking^b^		49 (26.6)	28 (30.1)	21 (23.1)	0.281
Use of immunosuppressive drugs^c,e^		8 (5.1)	6 (7.6)	2 (2.6)	0.157
Solid organ transplantation^c^		5 (3.1)	3 (3.6)	2 (2.6)	0.711
Acute COVID-19					
Cough^b^		133 (70.7)	61 (63.5)	72 (78.3)	0.027
Dyspnea^b^		151 (80.7)	70 (73.7)	81 (88.0)	0.013
Invasive mechanical ventilation^b^		34 (18.5)	0 (0.0)	34 (38,6)	< 0.001
Acute kidney failure^b^		13 (7.1%)	3 (3.1)	10 (11.4)	0.029
Length of stay, days		15,92 ± 17.7	9.56 ± 11.3	22,47 ± 20.6	< 0.001

MW: minimum wage (3 MW = R$ 613.50); and CVD: cardiovascular disease. ^a^Values expressed as n (%) or mean ± SD. ^b^Missing data ≤ 10%. ^c^Missing data between 10-20%.^d^Non-White: black (20.7%), brown (48.9%), and yellow (0.5%). ^e^Prednisone > 20 mg/day for more than two weeks, cyclosporine, cyclophosphamide, mycophenolate, rituximab, azathioprine, and/or chemotherapy in the last 30 days.

**Table 2:** Symptoms, spirometry, lung volumes, DL_CO_, respiratory muscle strength, and six-minute walk test 360 days after hospitalization for COVID-19 (D360) in the whole sample and by group (Ward vs. ICU).^a,*^

Variable	Total sample	Group		Crude p-value	Adjusted p-value**
		Ward	ICU		
	N = 189	n = 96	n = 93		
Follow-up time on the D360, days	363.9 ± 13.8	363,6 ± 13.6	364.2 ± 13.8	0.784	-
Symptoms of long COVID-19					
Cough^b^	35 (19.0)	18 (19.4)	17 (18.7)	0.907	0.443
Dyspnea^b^	80 (43.0)	38 (40.0)	42 (46.2)	0.397	0.274
Spirometry					
VC, L^b^	3.0 ± 0.8	3.1 ± 0.8	3.1 ± 0.8	0.934	0.145
VC, % pred^b^	90.1 [79.5-99.8]	92.8 [83.0-99.2]	87.0 [76.0-100.4]	0.285	0.931
VC < LLN, %^b^	40 (23.0)	16 (17.6)	24 (28.9)	0.076	0.229
FVC, Ls ^b^	3.0 ± 0.8	3.0 ± 0.8	3.0 ± 0.8	0.807	0.188
FVC, % pred^b^	86.8 ± 15.2	87.1 ± 13.7	86.4 ± 16.8	0.750	0.710
FVC < LLN^b^	55 (29.3)	24 (25.0)	31(33.7)	0.190	0.539
FEV_1_, L^b^	2.3 ± 0.6	2.3 ± 0.7	2.3 ± 0.6	0.508	0.247
FEV_1_, % pred^b^	83.0 ± 16.7	83.2 ± 17.1	82.8 ± 16.5	0.886	0.980
FEV_1_ < LLN^b^	65 (34.6)	32 (33.3)	33 (35.9)	0.715	0.635
FEV_1_/FVC^b^	77.5 [72.3-82.1]	77.9 [71.8-82.4]	77.3 [72.4-81.7]	0.695	0.913
FEV_1_/FVC < LLN^b^	84 (44.7)	42 (43.8)	42(45.7)	0.793	0.279
Lung volumes					
TLC, L^b^	4.7 ± 1.1	4.8 ± 1.0	4.6 ± 1.1	0.171	0.776
TLC, % pred^b^	87.2 ± 14.0	89.4 ± 13.5	84.8 ± 14.2	0.025	0.450
TLC < LLN^b^	50 (27.3)	17 (18.3)	33 (36.7)	0.005	0.085
RV, L^b^	1.6 ± 0.6	1.7 ± 0.5	1.5 ± 0.6	0.067	0.308
RV, % pred^b^	83.0 ± 25.5	86.9 ± 25.4	79.0 ± 25.1	0.039	0.253
RV/TLC, % pred^b^	96.9 ± 24.2	100.4 ± 23.9	93.2 ± 24.1	0.045	0.101
DL_CO_					
DL_CO_, mL.min^-1^.mmHg^b^	19.7 ± 5.4	20.1 ± 5.8	19.2 ± 4.9	0.284	0.690
DL_CO_, % pred^c^	93.1 ± 19.6	96.6 ± 19.9	89.4 ± 18.7	0.013	0.145
DL_CO_ < LLN^c^*	32 (17.8)	12 (12.9)	20 (23.0)	0.077	0.069
Respiratory muscle strength					
MIP, cmH_2_O^c^	76.6 ± 26.7	75.1 ± 28.9	78.2 ± 24.4	0.444	0.290
MIP, % pred^c^	85.5 ± 27.2	83.0 ± 27.7	88.0 ± 26.6	0.222	0.373
MIP < LIN^c^	35 (19.7)	20 (22.2)	15 (17.0)	0.385	0.297
MEP, cmH_2_O^c^	82.9 ± 30.2	81.0 ± 31.1	84.8 ± 29.3	0.400	0.578
MEP, % pred^c^	48.9 ± 16.3	48.4 ± 17.2	49.4 ± 15.4	0.680	0.788
MEP < LIN^c^	146 (82.0)	71 (78.9)	75 (85.2)	0.271	0.659
Six-minute walk test					
Distance, m^b^	486.4 [409.7-532.4]	492.2 [397.4-559.9)	466.0 [430.7-512.0]	0.099	0.220
Distance, % pred^b^	91.7 ± 17.1	94.0 [81.2-101.5]	90.0 [75.2-102.7]	0.368	0.820
Saturation drop during the test (ΔSpO_2_ ≤ 4%)^b^	53 (30.1)	25 (28.4)	28 (31.8)	0.622	0.685
HRR_1_, bpm^b^	91.0 [78.0-102.0]	91.7 ± 13.7	85.0 ± 19.3	0.616	0.990
Δ(final HR, HRR_1._), bpm^b^	22.7 ± 15.6	22.8 ± 15.7	22.7 ± 15.7	0.969	0.444
%HRmax^b^	71.3 ± 11.8	72.7 ± 11.9	69.9 ± 11.6	0.109	0.516
Final Borg scale score ≥ 4^b^	41 (23.3)	19 (21.6)	22 (25.0)	0.593	0.559

% pred: % of predicted values; LLN: lower limit of normality; HRR_1_: recovery heart rate in the first minute; and %HRmax: percentage of maximum HR achieved. ^a^Values expressed as n (%), mean ± SD, or median [IQR]. ^b^Missing data ≤ 10%. ^c^Missing data in 11-12%. *Variables expressed as median [IQR] were calculated with the nonparametric Mann-Whitney U test. **Adjusted for BMI, invasive mechanical ventilation, and length of hospital stay.

**Table 3:** Symptoms, spirometry, lung volumes, DL_CO_, respiratory muscle strength, and six-minute walk test 360 days after hospitalization for COVID-19 (D360) in the whole sample and by group (absence of dyspnea vs. presence of dyspnea).^a,*^

Variable	Total sample	Group		Crude p-value	Adjusted p-value**
		No dyspnea	Dyspnea		
	N = 186	n = 106	n = 80		
Follow-up time on the D360, days	363.9 ± 13.8	363,7 ± 13.4	364.3 ± 14.3	0.768	-
Symptoms of long COVID-19					
Cough^b^	35 (19.0)	11 (10.5)	24 (30.4)	0.001	0.010
BMI, kg/m^2^	32.3 ± 7.0	31.0 ± 6.4	34.1 ± 7.4	0.002	-
Spirometry					
VC, L^b^	3.0 [2.5-3.8]	3.3 [2.8-4.0]	2.7 [2.4-3.1]	< 0.001	< 0.001
VC, % pred^b^	90.1 ± 15.2	92.0 ± 14.8	87.3 ± 15.5	0.049	0.083
VC < LLN, %^b^	39 (22.8)	22 (21.6)	17 (24.6)	0.639	0.964
FVC, Ls ^b^	2.9 [2.5-3.6]	3.2 [2.7-3.8]	2.6 [2.3-3.1]	< 0.001	< 0.001
FVC, % pred^b^	86.8 ± 15.2	89.0 ± 15.0	83.8 ± 15.2	0.021	0.051
FVC < LLN^b^	54 (29.2)	25 (23.8)	29 (36.3)	0.065	0.077
FEV_1_, L^b^	2.3 ± 0.6	2.5 ± 0.6	2.0 ± 0.6	< 0.001	< 0.001
FEV_1_, % pred^b^	83.1 ± 16.8	86.6 ± 15.2	78.6 ± 17.7	0.001	0.001
FEV_1_ < LLN^b^	63 (34.1)	30 (28.6)	33 (41.3)	0.071	0.100
FEV_1_/FVC^b^	76.2 ± 9.1	77.5 ± 7.5	74.6 ± 10.6	0.027	0.002
FEV_1_/FVC < LLN^b^	81 (43.8)	46 (43.8)	35 (43.8)	0.994	0.576
Lung volumes					
TLC, L^b^	4.7 ± 1.1	4.9 ± 1.1	4.4 ± 1.1	0.001	0.012
TLC, % pred^b^	85.9 [79.6-95.2]	86.9 [79.9-95.2]	84.2 [77.4-95.0]	0.455	0.923
TLC < LLN^b^	49 (27.2)	27 (26.0)	22 (28.9)	0.657	0.718
RV, L^b^	1.6 ± 0.6	1.6 ± 0.5	1.6 ± 0.7	0.907	0.240
RV, % pred^b^	82.8 ± 25.5	80.3 ± 20.5	86.4 ± 30.9	0.115	0.035
RV/TLC, % pred^b^	97.0 ± 23.9	92.5 ± 22.0	103.0 ± 25.0	0.003	0.005
DL_CO_					
DL_CO_, mL.min^-1^.mmHg^b^	19.7 ± 5.4	20.9 ± 5.6	17.9 ± 4.7	< 0,001	< 0,001
DL_CO_, % pred^c^	93.0 ± 19.7	94.0 ± 19.3	91.6 ± 20.4	0.408	0.021
DL_CO_ < LLN^c^*	32 (18.1)	15 (14.9)	17 (22.4)	0.198	0.011
Respiratory muscle strength					
MIP, cmH_2_O^c^	76.1 ± 26.7	80.1 ± 28.6	70.9 ± 23.1	0.024	0.026
MIP, % pred^c^	85.0 ± 27.1	82.9 ± 26.9	87.9 ± 27.2	0.222	0.479
MIP < LIN^c^	35 (20.0)	22 (22.0)	13 (17.3)	0.445	0.750
MEP, cmH_2_O^c^	82.3 ± 29.8	85.8 ± 32.0	77.7 ± 26.2	0.077	0.085
MEP, % pred^c^	48.6 ± 16.3	48.0 ± 16.2	49.5 ± 16.4	0.568	0.912
MEP < LIN^c^	145 (82.9)	81 (81.0)	64 (85.3)	0.452	0.373
Six-minute walk test					
Distance, m^b^	486.4 [409.7-532.4]	512.0 [457.0-553.3)	440.3 [358.4-486.4]	< 0.001	< 0.001
Distance, % pred^b^	94.3 [82.7-103.2]	98.2 [87.8-105.4]	87.1 [75.4-99.3]	< 0.001	< 0.001
Saturation drop during the test (ΔSpO_2_ ≤ 4%)^b^	53 (30.6)	30 (29.7)	23 (319)	0.753	0.929
HRR_1_, bpm^b^	89.8 ± 18.7	91.7 ± 13.7	85.0 ± 19.3	0.118	0.062
Δ(final HR, HRR_1._), bpm^b^	22.7 ± 15.6	22.8 ± 15.3	22.7 ± 16.1	0.944	0.992
%HRmax^b^	71.3 ± 11.8	72.1 ± 11.5	70.1 ± 12.3	0.277	0.329
Final Borg scale score ≥ 4^b^	41 (23.7)	9 (8.9)	32 (44.4)	< 0.001	< 0.001

% pred: % of predicted values; LLN: lower limit of normality; HRR_1_: recovery heart rate in the first minute; and %HRmax: percentage of maximum HR achieved. ^a^Values expressed as n (%), mean ± SD, or median [IQR]. ^b^Missing data ≤ 10%. ^c^Missing data in 11-12%. *Variables expressed as median [IQR] were calculated with the nonparametric Mann-Whitney U test. **Adjusted for BMI, invasive mechanical ventilation, and length of hospital stay.

In the assessment of QoL, the group with dyspnea had worse mobility problems, self-care, usual activities, pain/discomfort, anxiety, and depression. The mean follow-up duration was 364 days (Table 4).

**Table 4:** Description of the dimensions of quality of life according to the EQ-5D3L questionnaire 360 days after hospitalization for COVID-19 in the whole sample and by group (absence of dyspnea vs. presence of dyspnea).^a^

Variable	Total sample	Group		p
		No dyspnea	Dyspnea	
	N = 186	n = 106	n = 80	
Mobility				
No problems	112 (60.2)	76 (71.7)	36 (45.0)	< 0.001
Some problems or inability	74 (39.8)	30 (28.3)	44 (55.0)	
Self-care				
No problems	155 (83.3)	100 (94.3)	55 (68.8)	< 0.001
Some problems or inability	31 (16.7)	6 (5.7)	25 (31.3)	
Regular activities				
No problems	132 (71.0)	88 (83.0)	44 (55.0)	< 0.001
Some problems or inability	54 (29.0)	18 (17.0)	36 (45.0)	
Pain/malaise				
Absent	78 (41.9)	58 (54.7)	20 (25.0)	< 0.001
Moderate/extreme	108 (58.1)	48 (45.3)	60 (75.0)	
Anxiety/Depression				
Absent	94 (50.5)	70 (66.0)	24 (30.0)	< 0.001
Moderate/extreme	92 (49.5)	36 (34.0)	56 (70.0)	
Comprehensive overview on health	80.0 [70.0 - 90.0]	87.5 [70.0 - 98.2]	80.0 [50.0 - 83.7]	< 0.001

a Values expressed as n (%) or median [IQR].

## DISCUSSION

The main results of this study show that dyspnea was present in 43% of the cohort at 12 months. Symptoms of cough and dyspnea in the acute phase predominated in the ICU group; however, at 12 months, there was no difference between the groups. About 27% of the cohort still had a restrictive ventilatory pattern, and 18% had altered DL_CO_ at 12 months. 

Corroborating our results, post-COVID-19 persistent symptoms were still observed in 30% of the subjects in the Wuhan cohort, China, at one-year follow-up, regardless of initial severity. These symptoms were related to decreased QoL, lower functional capacity, and abnormal mental health.^29^ A possible explanation for persistent dyspnea is a combination of peripheral and psychological factors.^30^

In another Brazilian cohort study, one year after hospital discharge, more than one-third of patients still had persistent COVID-19-related symptoms, regardless of acute disease severity. The most common symptoms were dyspnea (54.5%), fatigue (50.0%), myalgia, and muscle weakness (46.6%), which decreased over time. Obese patients also had a greater risk of dyspnea, although this was not significant after adjustment.^31^ In our dataset, BMI > 30 kg/m^2^ was not associated with persistent dyspnea.

According to plethysmography, pulmonary function still remained impaired in part of the cohort (27% restriction), regardless of the unit of admission (ward or ICU). After stratification by groups with and without dyspnea, reduced DL_CO_ was the only variable significantly associated with dyspnea at 12 months (p = 0.02). Huang et al.,^32^ when evaluating survivors of COVID-19 after 12 months, described 29% of restriction and 54% of altered DL_CO_ in individuals who required ICU admission. Meanwhile, Steinbeis et al.^33^ reported 44-50% restriction and 61-76% altered DL_CO_ among survivors who required high-flow oxygen and invasive mechanical ventilation, showing that these differences still persist after 12 months of follow-up.

Pulmonary fibrosis after COVID-19 may be related to restriction and altered DL_CO_ and may be explained by the duration of illness and mechanical ventilation use.^34^ Impaired DL_CO_ may also be attributable to vascular abnormalities.^35^ These data suggest that changes in lung function after 1 year are not enough to explain the late presence of dyspnea.

Regarding the exercise capacity assessment by 6MWT, individuals with persistent dyspnea walked a shorter distance (440.3 m vs. 512.0 m, p < 0.001). They also had a worse assessment of sensory stress (final Borg score ≥ 4). However, no differences were observed in gas exchange during exercise (desaturation ≥ 4%), in Δ(final HR, HRR_1_), suggesting that the worse 6MWD in individuals with persistent dyspnea may be due to the peripheral muscle component. Razak et al.,^2^ in their analysis of 119 survivors of COVID-19 in 12 months, also justified the shorter 6MWD in their patients as a result of muscle weakness.

The data presented here have shown that some mobility problems, and anxiety/depression were present in more than 50% of the individuals with dyspnea. Similarly to our results, Schlemmer et al.^36^ found that although most participants recovered overall, high percentages had functional sequelae and residual symptoms over the course of follow-up, all of which may have affected their HRQoL.

Evidence is insufficient to determine conclusions about the underlying mechanisms of post-COVID breathlessness. A previous review study reported inconsistent results of impaired lung function or lung pathologies, although correlations between mental health disorders (depression and anxiety) and post-COVID-19 breathlessness appear to be more consistent.^37^ Sakai et al.^38^ suggest that rehabilitation after COVID-19 should be considered an effective therapeutic strategy to improve the functional capacity and QoL of patients with COVID-19.

The strength of our study is its multicenter design, in which regional public referral hospitals for the treatment of patients with COVID-19 participated. Specialized trained teams, including undergraduate and graduate students, as well as research professors, carried out data collection systematically and were able to identify patients according to their disease severity using a standardized questionnaire through REDcap.

This study has limitations. There was no information on return to work, use of health care services, and mental health status of the patients after discharge. Therefore, longitudinal analysis of these outcomes was not possible. Similarly to most COVID-19 follow-up studies, there is a potential information bias regarding self-reported comorbidities during the acute phase and during convalescence. The outcome of patients who missed follow-up and were not assessed 1 year after admission is unknown, and most of the patients were those who had been admitted to an ICU.

Twelve months after acute infection, survivors of severe COVID-19 still had a high burden of symptoms, such as dyspnea, restrictive ventilatory changes in lung function, and loss of QoL, identified in an established cohort in a middle-income country that had been highly impacted by the pandemic.
